# The Influence of Different Length Aluminum Foam Filling on Mechanical Behavior of a Square Thin-Walled Column

**DOI:** 10.3390/ma14133630

**Published:** 2021-06-29

**Authors:** Michał Rogala, Mirosław Ferdynus, Katarzyna Gawdzińska, Paweł Kochmański

**Affiliations:** 1 Department of Machine Design & Mechatronics, Faculty of Mechanical Engineering, Lublin University of Technology, Nadbystrzycka 36, 20-618 Lublin, Poland; m.ferdynus@pollub.pl; 2 Faculty of Marine Engineering, Maritime University of Szczecin, Willowa 2-4, 71-650 Szczecin, Poland; k.gawdzinska@am.szczecin.pl; 3 Faculty of Mechanical Engineering and Mechatronics, West Pomeranian University of Technology, al. Piastow 19, 70-310 Szczecin, Poland; pawel.kochmanski@zut.edu.pl

**Keywords:** energy absorber, thin-walled structures, dynamic crush, foam filling, aluminum foam, porous structures

## Abstract

The demand for lightweight, strong structural profiles is currently high in the transport industry, mechanical engineering, and construction. Therefore, it is important to evaluate their properties, especially mechanical properties. The main objective of this paper is to determine energy absorption coefficients and evaluate the crush resistance of thin-walled aluminum profiles using numerical simulation and empirical verification. This paper presents the compression results of testing of thin-walled aluminum profiles filled with a porous material (cast aluminum foam). The numerical analysis was conducted using the software Abaqus/CAE. Aluminum material data were obtained from a static tensile test performed on a Shimadzu machine. The experiment was performed on an Instron CEAST 9450HES dynamic hammer. Profiles with three shapes of crush initiators filled with aluminum foam measuring 40 mm–200 mm in 20 mm increments were numerically tested. A sample with a concave initiator filled with foams of 40 mm, 60 mm, 80 mm, and 120 mm in length was used to verify the numerical analyses. Energy absorption coefficients were determined from the analyses. The results of both analyses were tabulated to show the percentage differences. The study showed an increase in the Crush Load Efficiency (CLE) index by up to 33% for samples with the same crush initiator. In addition, it was noted that the use of porous fill does not increase the value of initiating Peak Crushing Force (PCF), which indicates the generation of much smaller overloads dangerous for vehicle passengers.

## 1. Introduction

In the automotive industry, user safety remains an important factor in vehicle design. As early as the beginning of the 20th century, the first solutions were used to protect the main components of the vehicle as well as the occupants in the vehicle. In the 1960s, the first regulations defining safety considerations in vehicles appeared [1].

Initially, the protective element was a bumper made of a flat steel bar attached to the front supporting parts of the vehicle. Over time, the design of this area of the vehicle began to change, plastic bumpers and foam inserts appeared, which had energy-absorbing properties [2]. In the 1990s, additional energy-absorbing elements called crash-boxes appeared in the design of the crumple zone in the form of thin-walled metal structures mounted on the stringers. The purpose of crash-boxes is to absorb a large portion of the energy and protect the stringers, which are extremely expensive to repair, during collision speeds of up to 15–20 km/h.

Manufacturers of new vehicles have to meet increasingly stringent requirements, which often seem to be contradictory. On the one hand, it is necessary to maximize the amount of energy absorbed by the crush zone, and on the other hand, not to generate large overloads, which is related to the occurrence of PCF (Peak Crushing Force) at the beginning of the crush [3]. To meet these requirements, designers experimented with thin-walled profiles with different cross-sections, which were often adapted to the cross-sectional shape of the stringer. The first scientific papers on these problems appeared [4]. In the 1980s, Abramowicz worked on the study of axial dynamic crushing in both square and circular section models [5,6] using analytical and experimental considerations. Specimens differing in the ratio of wall thickness to profile height were analyzed. Hanssen et al. [7,8] were among the first researchers in the area of foam-filled energy absorbers. The papers presented considerations on how to model the foam issue and the energy absorption of aluminum fillings with different densities under static and dynamic loading. Kaczynski et al. presented a quasi-static and dynamic study of energy absorption by aluminum and composite foams depending on their manufacturing technology [9]. The protection of passengers (and cargo, environment) is also not without significance, e.g., protection from leakage of photovoltaic cell batteries located in the chassis of motor vehicles, and other related issues to fire protection. For example, design principles are described due to thermal and mechanical actions on structures under fire conditions. Fire-resistant aluminum structures can also be helpful, especially aluminum profiles and foams. Hipke et al. in [10] presented an interesting study on layered aluminum foam core materials. They described the high stiffness of these structures with low weight and high fire resistance. They proved that these materials protect electromagnetic waves and show significant vibration reduction and acoustic isolation. In one of the authors’ works [11], a study of the fire resistance of aluminum and aluminum-ceramic foams is presented.

Porous structures also find their application during the study of sandwich-type structures [12]. Metal or metal-ceramic foams are covered with plate elements that havedifferent mechanical properties. By using foam filling, a significant increase in the absorbed energy can be observed in the three-point bending test. An interesting approach in the study of porous materials was presented by Duarte et al. [13], where the open-pore foam was flooded with a polymer. The filling formed a composite structure, the effect of which positively affected the energy absorption performance. However, the authors noted that filling a thin-walled profile fully causes damage to the aluminum column during dynamic testing due to too much polymer inside. Many works investigating porous fillings focus on changing the cross-sectional area through various types of cut-outs [14,15]. The authors present the concept of cut-outs with bio-inspired shapes such as squares along with the profile (cornstalk). All the literature aptly considers reducing the amount of porous material; however, none have researched the area of reduction in fill length and its effect on crush efficiency indices.

At the beginning of the 21st century, a theoretical work [16] was presented that focused on the failure of models, both multi-cell and those with foamed material filling [17]. In modern designs, energy absorbers are usually made with a so-called crush initiator called a trigger. Its task is to induce the start of the plastic process in a precisely defined place. Many studies [18,19], as well as works of the authors [20,21], show that the presence of the trigger significantly reduces the PCF value and thus improves the CLE index (Crush Load Efficiency); however, it is accompanied by a smaller portion of energy absorbed during the generation of the first fold. Therefore, the idea of partial foam filling, which is the subject of this article, was developed to improve energy absorption without significantly changing the performance characteristics, as is the case with “full” filling. The use of foam with an appropriate density does not significantly increase the value of PCF, but it dramatically improves the energy-absorbing properties by increasing the MCF (Mean Crushing Force).

In many studies, the authors focused on maximizing the amount of energy absorbed by a thin-walled structure concerning its SEA mass; in this case, it was easily achieved by using multi-cell [22,23] or bi-tubular [24,25] thin-walled profiles, also filled with foam of an appropriate density [26,27] or honeycomb [28,29,30] structures. The use of multi-cell structures is a solution that is becoming more popular; however, such solutions do not usually fulfill an important condition set for biomechanical reasons, namely the minimization of overload that occurs during crushing.

As mentioned above, a method to avoid the increase of PCF force while increasing the energy efficiency of the energy absorber is to use foamed material fillings; this is especially true for mono-cell profiles, which are characterized by lower values of PCF forces compared with multi-cell profiles.

The high energy performance of thin-walled structures is a result of the mechanism of crush progression. Local loss of stability occurs in areas of imperfection, which appear naturally or are forced by geometric disturbances in the form of a crush initiator. In literature, many positions describe the deformation process of the thin-walled model, of which the so-called progressive crush is the most desirable [31]. In order to obtain this form of deformation in the thin-walled structure, apart from the essential dimensions of the energy absorber, especially its slenderness, important factors include the presence, location as well as shape of the crush initiator [28]. When analyzing issues with many factors whose significance and influence are difficult to grasp, the use of artificial intelligence methods seems unrivaled. During regression analyses, it is possible to find relations between input data, e.g., geometrical variables related to the shape and position of the crush initiator, and output data, i.e., crush efficiency indexes [32,33]. It is also possible to predict the values of these parameters for obtaining desired energy-absorbing properties, which significantly reduce the time and cost of calculations.

## 2. Crashworthiness Indicator

**Figure 1:**
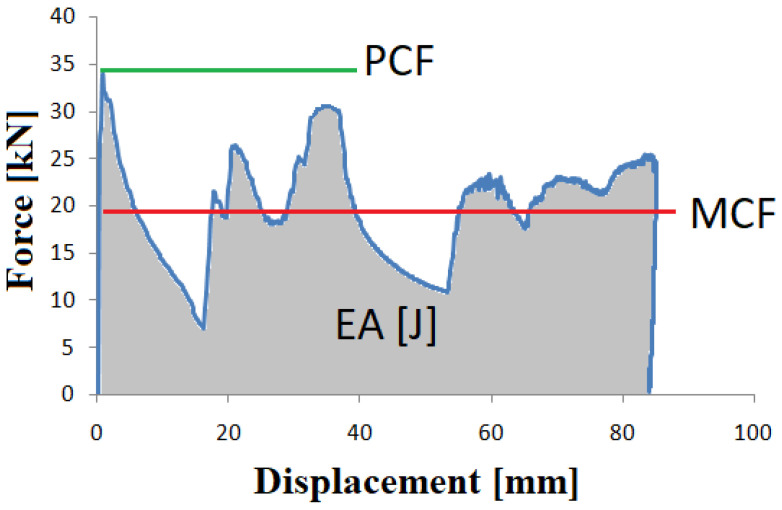
Example of force-displacement characteristics of an energy absorber.

The basic quantity determined during analysis was the amount of mechanical energy dissipated during the dynamic crushing process. Its measure is the area under the curve shown in Figure 1 and is given by the formula:

(1) $$EA=\int_{0}^{d}F\left(x\right)dx,\;\left[J\right]$$

The value of this energy is generally related to the mass of the energy-absorber, which is denoted as *SEA* (Specific Energy Absorption), and is given by the formula below:

(2) $$SEA=\frac{EA}{m},\;\left[\frac{J}{kg}\right]$$

Another indicator is the Mean Crushing Force—*MCF* (Figure 1), which occurs during crushing and is defined as the ratio of energy absorbed to total crushing distance:

(3) $$MCF=\frac{EA}{d_{x}},\;\left[N\right]$$

where:

*EA*—energy absorbed;

*d_x_*—crushing distance.

An important parameter is the Peak Crushing Force (*PCF*) value, which is shown in Figure 1. The value of this force directly affects the maximum overloads that occur at the beginning, and their duration will determine the probability of severe injury and the chances of survival.

The ratio of the average crush force *MCF* to the maximum crush initiating force *PCF* is referred to as the *CLE* ratio and is given by the equation:

(4) $$CLE=\frac{MCF}{PCF}\left[-\right]$$

For biomechanical reasons, it is advantageous to crush as long a displacement as possible; hence the SE ratio of displacement to initial specimen height:

(5) $$SE=\frac{U}{L_{0}},\;\left[-\right]$$

where:

*L*_0_—initial specimen length [mm];

*U*—maximum specimen shortening (crushing distance) [mm].

The last indicator that comprehensively shows the performance of the energy absorber is the Total Efficiency (*TE*), and it is represented as the product of the indicators in Equations (4) and (5).

(6) TE=CLE×SE [−]

## 3. Materials and Models

**Figure 2:**
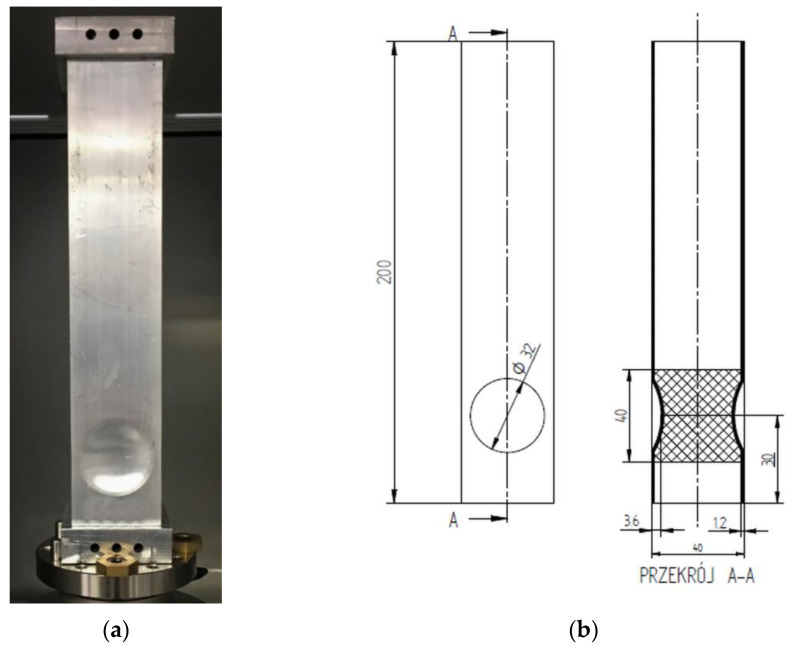
(**a**) Physical view of the specimen attached to the test table; (**b**) The technical drawing with dimensions of the C32 model filled with 40 mm length foam.

The subject of the analysis was a profile with a square cross-section. The model was filled with a porous aluminum material of different lengths. The dimensions of the aluminum profile were 200 mm high, a cross-section of 40 × 40 mm, and a sidewall thickness of 1.2 mm (Figure 2). The length of the foam fill occurred from 40 mm to 200 mm every 20 mm. The aluminum foam was manufactured as a block ex situ. Then, after cutting to the specified size, it was pressed inside the profile. Using such technology made it possible to obtain a structure with identical properties in each of its places, and the pores produced reached similar sizes for the whole structure.

The material model of the aluminum column was defined as elastic-plastic. Currently, for dynamic problems, the Johnson–Cook constitutive material model considers the strain rate as most commonly used [34]. In many works, this decision was justified by showing large discrepancies for different velocities of the given load, especially during the explosion study. However, based on Wizner’s research [35], aluminum alloys with T6 heat treatment show that load dynamics do not affect the strain rate. Given these results, the model was simplified to minimize the computational cost. The material data of EN AA6060-T6 aluminum was obtained by static tensile testing. The elastic properties were based on Young’s modulus and Poisson’s ratio, where as the plastic properties were based on multi-linear characteristics of the stress-strain relationship. The obtained data are shown in Table 1.

**Figure 3:**
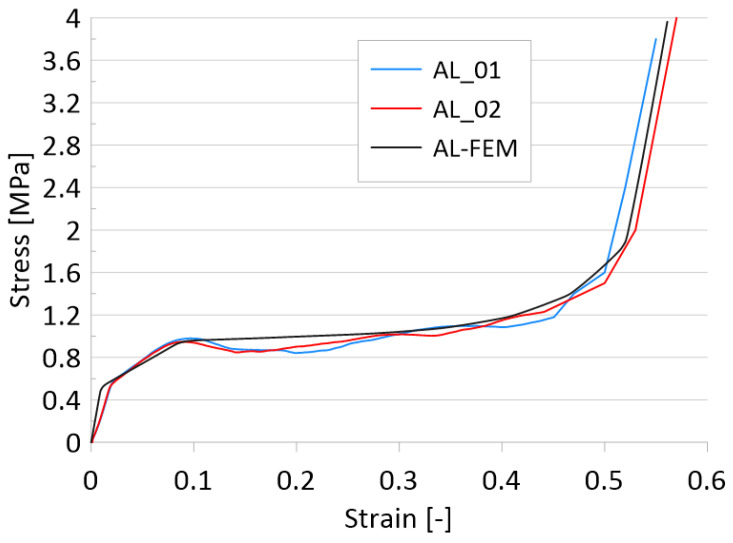
Stress-strain curve of the foamed material.

The foam material in the plasticity field was modeled as Crushable foam [36]. The necessary data have yield strength in uniaxial compression *σ*^0^*_c_*, initial yield stress in hydrostatic compression *p*^0^*_c_*, yield strength in hydrostatic tension *p_t_*, and plastic Poisson’s ratio *ν_p_*. The foamed material data are shown below in Table 2 and Figure 3.

The results of the test presented above were obtained using a Comatech test machine. The dimensions of the sample were determined based on the literature position [38], where the author described the relationship between the height, width, and length of the sample. The aluminum foam was cut to the size of 40 × 40 × 20 mm. The speed of the compression plates was determined to be 2 mm/min. The test was repeated twice, and the result was compared with FEM analysis to validate the material model.

In order to validate the provably modeled porous material, a cubic sample was subjected to a compression test. The characteristics in Figure 3 show two experimental specimens labeled AL_01 and AL_02. The curve obtained from the numerical analysis is AL-FEM and reflects high accuracy, confirming the correct modeling of the porous structure.

The modeled material described above is based on constitutive equations, where the yield surface for isotropic hardening can be described as follows:

(7) $$F=\sqrt{q^{2}+\alpha^{2}p^{2}}-B=0$$

where:

(8) $$p=-\frac{1}{3}trace\;\sigma ,\;\;\;\;\;\;\;\;\;q=\sqrt{\frac{3}{2}\sigma_{dev}:\sigma_{dev},}$$

*p*—pressure stress, *q*—von Mises stress.

Parameter *B* is the size of yield ellipse related to the hydrostatic compression *p_c_*:

(9) $$B=\alpha p_{c}=\sigma_{c}\sqrt{1+{\left(\frac{\alpha }{3}\right)}^{2}}$$

The yield surface represents the Mises circle in the deviatoric stress plane. The shape factor, α, can be computed using the initial yield stress in uniaxial compression *σ*^0^*_c_*, and the initial yield stress in hydrostatic compression, *p*^0^*_c_* (the initial value of *p_c_*), using the dependencies:

(10) $$\alpha =\frac{3k}{\sqrt{9+k^{2}}}\;\;\;\;\;\;\;\;with\;\;\;\;\;\;\;\;k=\frac{\sigma_{c}^{0}}{p_{c}^{0}}$$

## 4. Methodology of FEM

**Figure 4:**
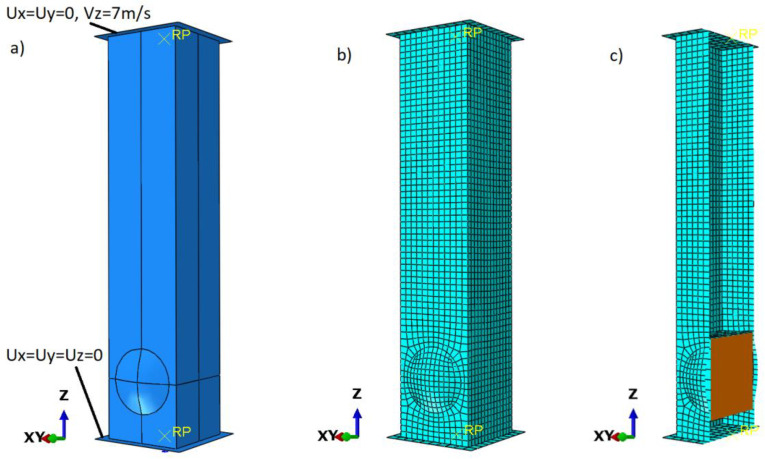
Numerical model (**a**) Boundary conditions; (**b**) discretized numerical model; (**c**) cross-section of the discretized foam-filled model.

The analysis for each model was performed in two steps. The first was a buckling analysis, which was implemented into the dynamic analysis. The boundary conditions were reduced to those prevailing during the experimental study. The model was restrained by creating a non-deformable plate to which the aluminum profile was connected by a Tie relation. The load was modeled by assigning mass to the non-deformable plate and defining its initial velocity afterward (Figure 4). The modeled foam was connected with the profile only when friction force was applied as a contact definition. The friction ratio was set as 0.2.

**Figure 5:**
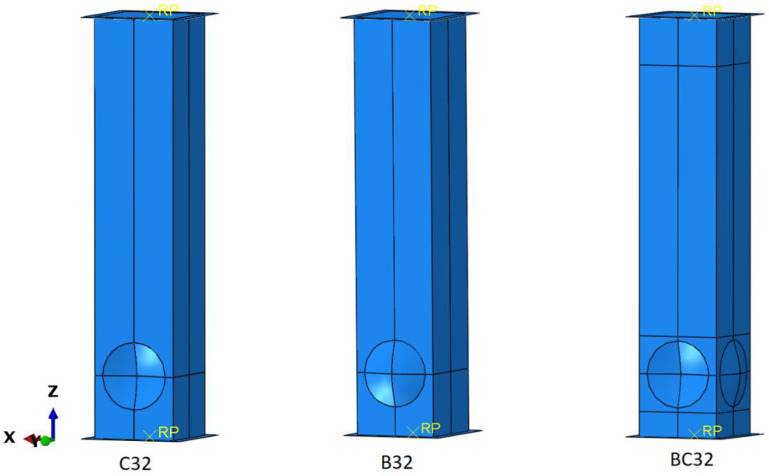
Types of models used in the numerical analysis.

The mass assigned to the plate was 70 kg, and its velocity was 7 m/s, which provides a kinetic energy of approximately 1700 J. The analysis of the problem was based on three geometric models with circular crush initiators. The first imperfection was a concave surface on the opposite side, with a diameter of 32 mm, and an indentation depth of 3.6 mm. The distance from the center of the circle to the bottom edge of the model is fixed at 30 mm. This model is referred to as the letter C in the results (Figure 5). The second type of initiator has a convex surface on its side with the same dimensions as in model C. During data analysis, the model was denoted by the letter B. The last model has convex surfaces on opposing faces and concave surfaces on the other two faces. It has geometric parameters as in the previous models and is denoted by the letters BC.

## 5. Experimental Studies

**Figure 6:**
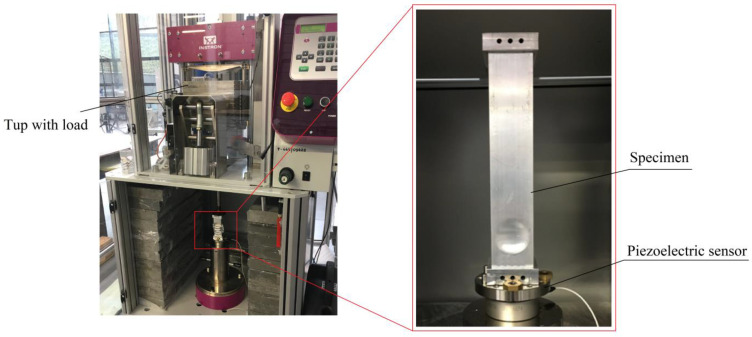
General appearance of the experimental test station and specimen location.

The impact test was performed on an Instron CEAST 9350HES hammer, the general appearance of which is shown in the image below (Figure 6). The stand consists of a test table onto which the specimen is attached. In addition, the dynamic testing hammer has a tup to which the mass is applied, thus translating into the energy obtained.

The fixing of the sample was done with dedicated aluminum cubes whose dimensions were matched to the internal dimensions of the profile. The purpose of the grips was to stabilize the specimen during crushing and properly translate the impact of the tup into the crushing of the absorber.

### 5.1. Foam Fabrication

**Figure 7:**
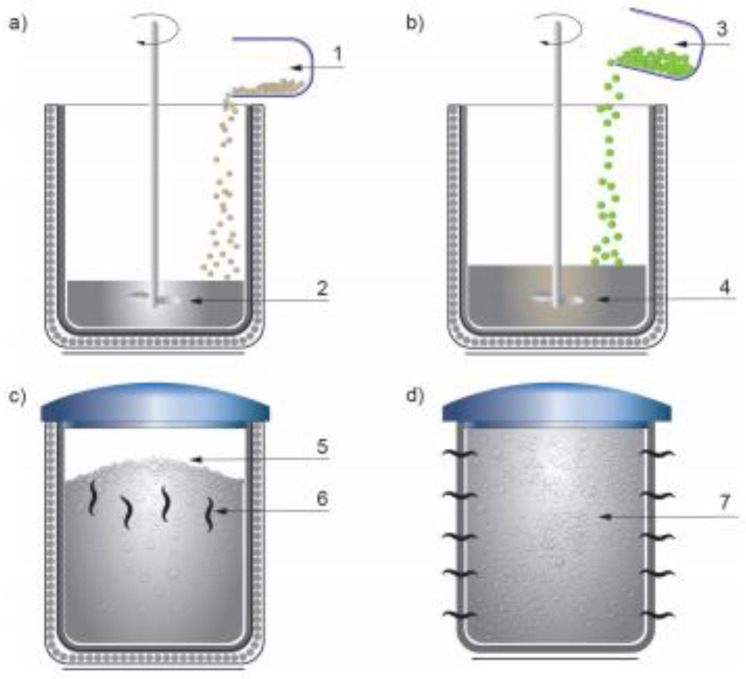
Scheme of the foaming method in the liquid state: (**a**) thickening, 1—Ca additive, 2—liquid aluminum; (**b**) 3—foaming agent TiH2, 4—thickened aluminum; (**c**) foam formation as a result of the chemical reaction, 5—foamed aluminum, 6—foam drainage; (**d**) foam cooling, 7—finished product of aluminum foam (own elaboration based on [39,40,41]).

In this paper, porous structures with density 0.292 g/cm^3^ and porosity 91.02% were used for foaming in a liquid state. Foaming in a liquid state implements a foaming agent in the form of a TiH2 compound into liquid aluminum thickened with calcium. As a result of chemical reaction, the foaming of liquid aluminum occurs. After adequate cooling of the foam, the finished product is obtained. The scheme of the method is shown in Figure 7.

This method is the most common way of manufacturing metal foams and allows the shaping of finished products during the technological process with repeatable structure parameters [39,40,41,42,43]. The foam produced was subjected to SEM-EDS analysis which showed the percentage (mass) of elements for the selected points. The averaged values were 81.2% Al, 4.1% Ca, 6.3% Ti, 8.0% Fe, and 0.4% Ni, respectively.

### 5.2. Experimental Specimen

**Figure 8:**
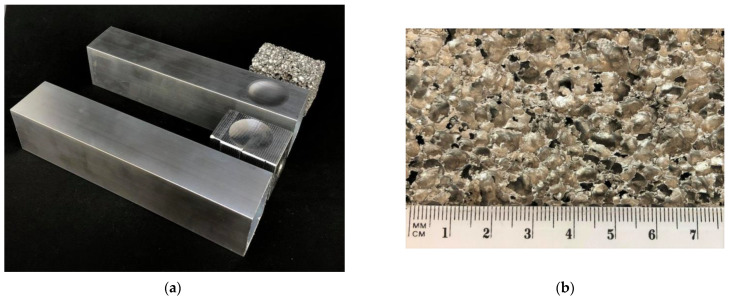
(**a**) The shape of the experimental specimen with matrix and foam filling presentation; (**b**) close-up of porous structure.

A model with a concave crush initiator described as C32-3 was experimentally tested on. Five experimental tests were performed. The specimens included a C32 empty profile and four aluminum foam-filled models with lengths of 40 mm, 60 mm, 80 mm, and 120 mm. The thin-walled models were made of EN AA6060-T6 aluminum alloy. The cross-section size was 40 mm × 40 mm, with a wall thickness of 1.2 mm. Profile height was 200 mm. A crush initiator was embossed on the surfaces of two sides using a hydraulic press and a dedicated die. The shape of the indentation is shown in the following graphic (Figure 8a), and corresponds to the initiator on the numerical model.

Figure 8a, shown above, figuratively describes the fabrication of a thin-walled specimen with a round trigger stamped on it. The dedicated die adjacent to the profile on the left consists of two parts fastened together with a screw. It is inserted into the center of the profile, and the initiator shape is then embossed using a hydraulic press and special rubber. Finally, aluminum foam (Figure 8b) of a specified length (60 mm in the picture) is pressed into the profile.

## 6. Results of Experimental Study

**Figure 9:**
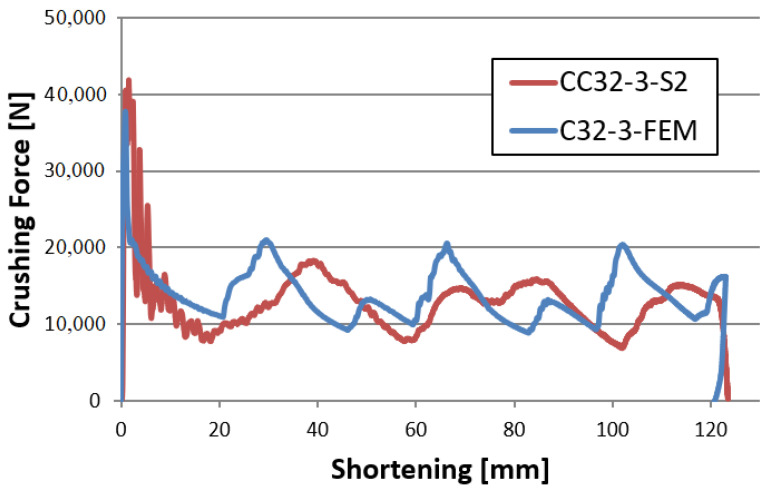
The force-shortening curve for an empty model with a concave crush initiator.

**Figure 10:**
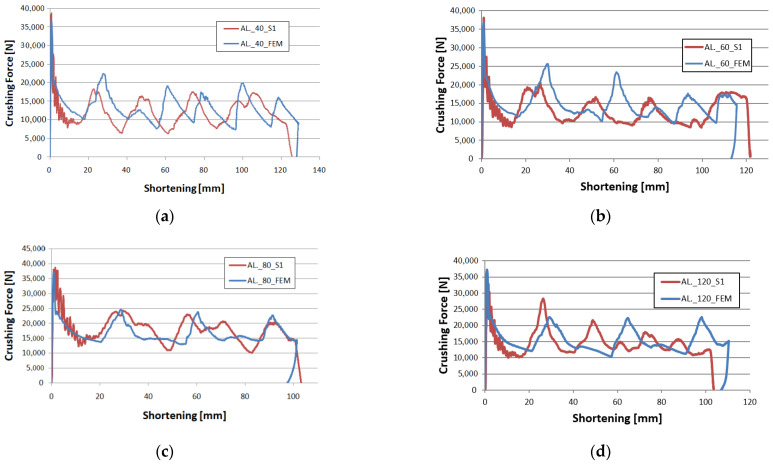
Experimental and numerical force-shortening diagram: (**a**) column with 40 mm foam filling; (**b**) column with 60 mm foam filling; (**c**) column with 80 mm foam filling; (**d**) column with 120 mm foam filling.

In the experimental analysis, 40 mm, 60 mm, 80 mm, 120 mm foamed samples and an unfilled control sample were tested. All the experimentally tested specimens had an initiator in the form of a concave surface denoted by the letter C in the numerical studies. Figure 9 and Figure 10 show the characteristics of the force-shortening relationship. The results are compared with the curve obtained during the numerical analysis.

The above graph compares numerical and experimental characteristics of samples without filling with a porous material. It functions as a control absorber to which the foam-filled profiles are compared. Based on the characteristic curves and the crushing efficiency ratios, it is possible to determine the influence of the porous structure on the absorber performance. The PCF occurs at the beginning of the analysis and reaches a value of approximately 40 kN. On the graph, force peaks can be seen, which correspond to the formation of plastic hinges whose number and occurrence in numerical and experimental analyses are similar, proving the convergence of both analyses.

Figure 10a–d compares force-shortening characteristics for the experimental and numerical analyses. In the graphs, we can see the shortening of the profile during dynamic crushing as well as the force values. The changing force indicates the formation of plastic hinges on thin-walled profiles. In the crush analysis, it is important to note how many plastic hinges were formed and in which places. A correlation of force peaks can be seen in the graphs, indicating that the absorbers behave similarly. In the case of the 120 mm foam sample, the last force peak splits into two smaller peaks. This behavior in the models may be due to material inhomogeneity or imperfections in the geometric structure of the specimen. However, this does not translate into crush efficiency ratios as both MCF and CLE values remain similar to numerical data.

**Figure 11:**
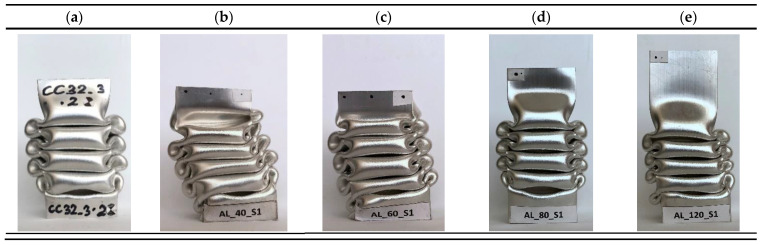
Comparison of experimentally tested specimens with foam length (**a**) 0 mm, (**b**) 40 mm, (**c**) 60 mm, (**d**) 80 mm, and (**e**) 120 mm.

All specimens were tested at the same initial energy, i.e., 1700 J, and the analysis was conducted until the value of the crushing force decreased to zero, i.e., until the total energy was absorbed by the profile. Testing at these initial conditions allows us to graphically determine the effect of porous material on the performance of the energy absorber from Figure 11. By observing successive profiles, one can see that the increasing height of the profile indicates that the same portion of energy was absorbed using a smaller length of the absorber. The higher the profile is, the greater the potential for the thin-walled structure to dissipate additional energy. Due to the skewness of specimens AL_40 and AL_60, which suffered a non-axial impact, an increase in specimen height with increasing foam insert length was not observed in this case.

Based on the obtained performance indicators and the course of force values, the numerical model was considered, validated and its reliability was confirmed.

**Figure 12:**
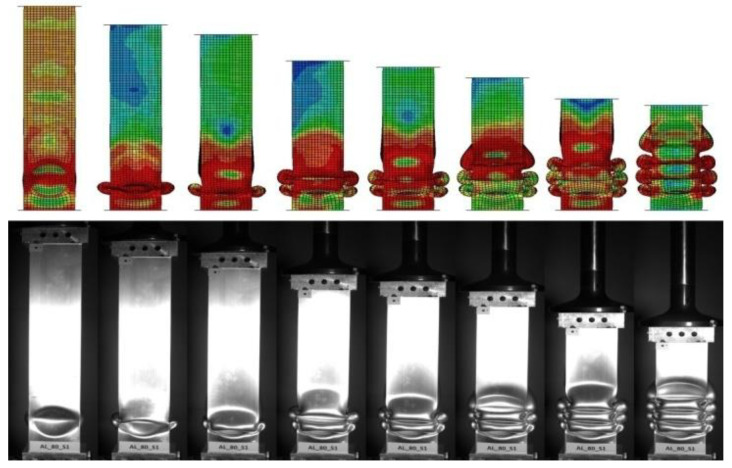
Comparison form of crushed absorber in different stages of dynamic analysis for 80 mm length foam.

**Figure 13:**
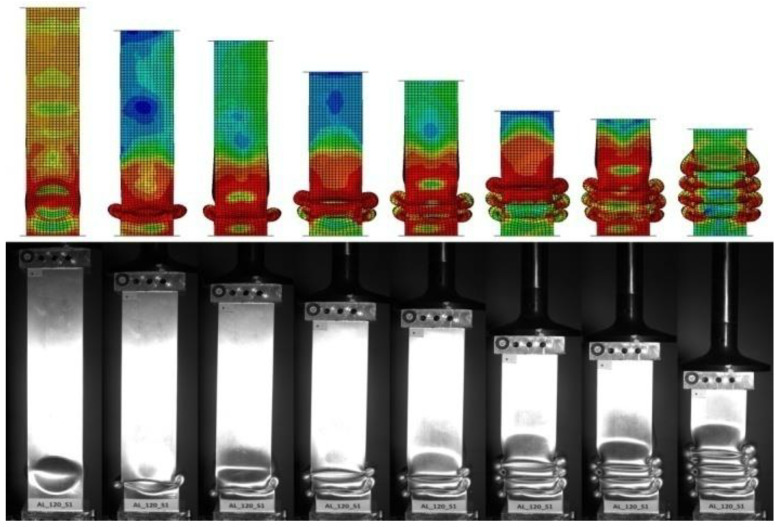
Comparison form of the crushed absorber in different stages of dynamic analysis for 120 mm length foam.

During the experimental analysis, the duration of the dynamic process (approximately 40 ms) required the use of a high-speed camera. The time-lapse video can map the behavior of the thin-walled experimental specimen and then compares it with visualizations from numerical analyses. Images of characteristic moments of the dynamic crush, i.e., formation of consecutive folds, are shown in Figure 12 and Figure 13.

The camera was used to analyze two foam-filled samples measuring 80 mm and 120 mm in length. The specimens had markers to obtain the change in displacement and velocity through time. By acquiring data from the high-speed camera, we can compare the efficiency indicators with those obtained numerically and determine the discrepancy between the two analyses.

**Figure 14:**
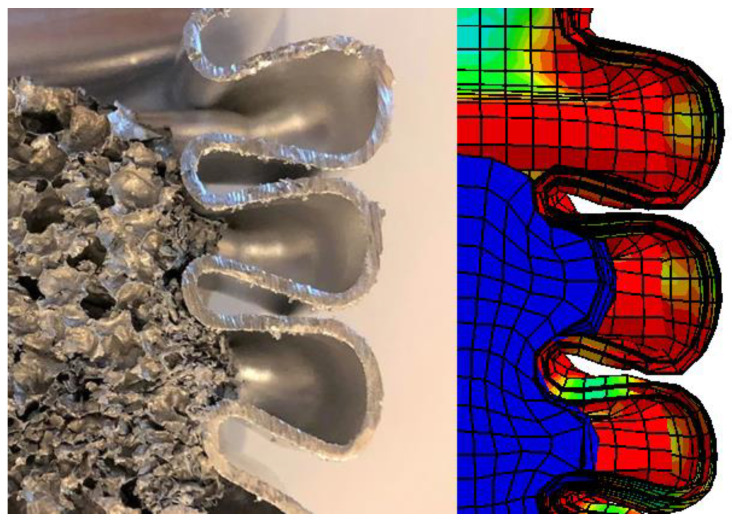
The shape of plastic hinges for a 60 mm long aluminum foam-filled specimen.

The graphic above (Figure 14) compares the cross-section of AL._60_S1 and AL._60_FEM. From the graphic, we can identify the shape of the plastic zones and their occurrence, as well as the behavior of the porous filling. The aluminum foam similarly fits into the folds and then deforms under the pressure of the aluminum profile walls. This process demonstrates a properly simulated material model and the manner in which the issue was modeled.

## 7. Results of Numerical Analysis

**Figure 15:**
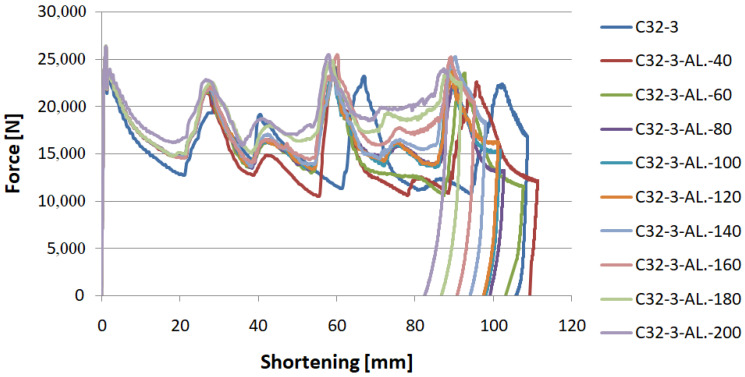
Force-shortening curves with different foam filling on an example of BC trigger.

The tested models were divided into three groups due to the shape of the crush initiator. The aluminum profiles filled with foamed material were subjected to dynamic crushing with an initial energy provided of 1700 J. Figure 15 shows the runs for all filling lengths for the concave-convex trigger denoted by BC.

**Figure 16:**
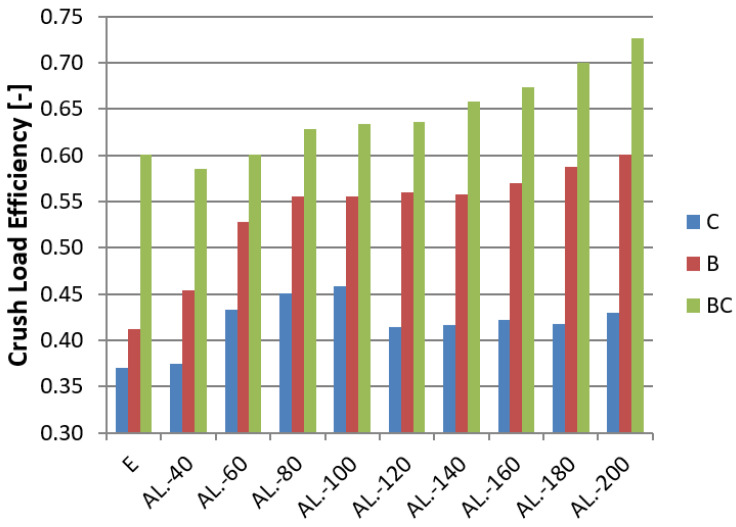
CLE indicator for all tested absorbers.

The results were based on the indices described in Section 2, presented with Formulas (1)–(5). The most important index characterizing the energy absorber is the Crush Load Efficiency (CLE) presented in Figure 16.

**Figure 17:**
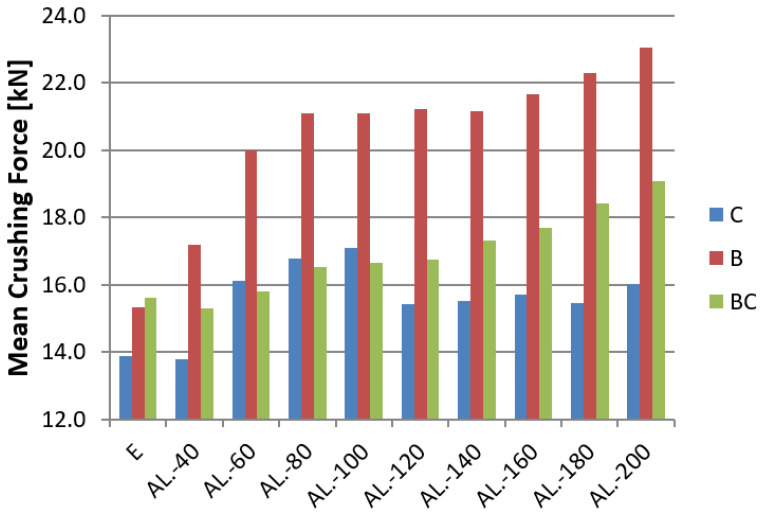
Mean Crushing Force for whole length range of aluminum foam filling.

**Figure 18:**
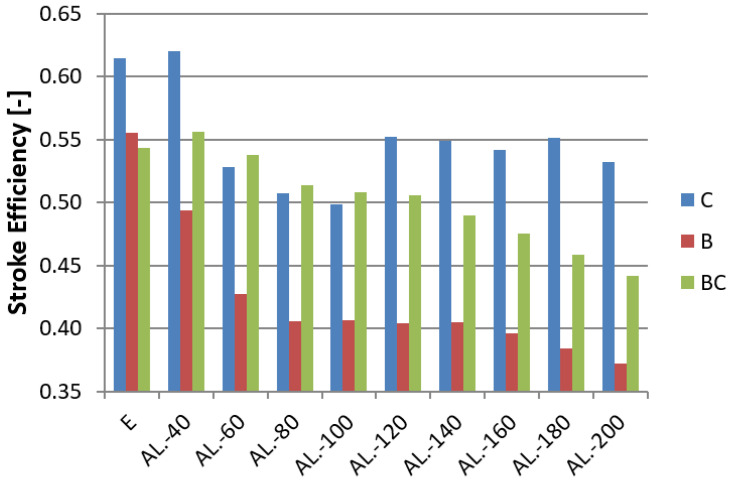
SE indicator for specimen filled with aluminum foam.

In the figures for each model (Figure 16, Figure 17 and Figure 18), a specific type of trigger is presented, and the sample without foam filling is marked with the letter E (Empty). Observing the CLE coefficient, the influence of foam filling on its increase is noticeable. The highest increase is observed for models with a convex crush initiator. In the case of models B and C, a clear rise in performance can be seen for foams measuring 60–100 mm in length. Models with this foam range have the best capacity considering the amount of porous material used. Longer fill lengths marginally increase efficiency, and in the case of model C, there was none at all. For the BC model, due to the nature of the triggering operation, the foam was crushed at the initial stage, resulting in little change in the CLE rate for foam fill lengths less than 100 mm. The MCF (Figure 16) value increases in a similar manner to the CLE index because of the value of the maximum force, which varies within a small range for individual initiators.

The average force increases with foam length for all trigger shapes. The jumping trend can be witnessed, especially for the 60–100 mm range where the increase in value is highest.

The ratio of SE decreased by about 15–20% for extreme cases. The difference is visible, especially for the B and C models (Table 3). The smaller the SE ratio, the better the potential for energy absorption for a profile of the same length.

The results presented in Table 4 show the values of the crush efficiency ratios for the numerical and experimental models verified by the dynamic machine. The values for each specimen are tabulated, and the percentage differences are presented. Among the models tested, the largest discrepancy did not exceed 8%, and for most of the data, the approximate difference was up to 4%.

## 8. Conclusions

This paper presents a numerical and experimental analysis of the crushing of thin-walled aluminum profiles partially filled with aluminum foam. Our research has shown the positive influence of the porous structure on crush efficiency indicators. The interaction of three types of triggers with aluminum foam was studied, where the profiles with a concave-convex initiator, described as BC in Figure 16, Figure 17 and Figure 18, showed the best alignment. The most effective foam performance was seen for lengths in the 80–120 mm range, particularly for models with a concave initiator, which was experimentally verified. Numerical and experimental results presented in the form of force-shortening diagrams showed significant agreement in terms of the course and value of maximum shortening. Correspondence between the deformation process in the experiment and that obtained in the numerical FEM simulation was also confirmed by the analysis of high-speed camera imagery (Figure 12 and Figure 13).

Based on the numerical data, one can observe a significant effect of the trigger’s configuration (CB vs. BC) on the magnitude of the PCF; however, this value hardly changes at all for different fill lengths with the porous material. Nevertheless, a significant effect on MCF and CLE indices was observed. The increase in their values for individual triggers reached up to 30–50%, proving the positive influence of specific porous fill lengths while keeping the PCF at the same level. Due to the low density and high strain rate in this analysis, the foam filling absorbs the most energy in the last stage of crushing. The filling was too weak to change the value of PCF; nonetheless, as the crushing process progressed, the foam started to absorb increasingly more energy, which is particularly noticeable in Figure 15.

It was noticed in the results that the final value of TE for models with the same crush initiator is practically invariant; this is due to the initial assumptions for the tests performed, where the specimens were subjected to this energy. Since the foam insert absorbs more energy the longer it is in length, it was accompanied by a decreased value of shortening and therefore a lower value of SE. TE, as a product of CLE and SE, remained practically unchanged because as the length of the foam insert increased, CLE increased, and SE decreased simultaneously. Thus, calculating TE when considering a foam insert is not authoritative.

## Figures and Tables

**Table 1:** Material properties of aluminum (own research).

AA.—6060-T6		Stress σ [MPa]	Strain ε [‒]
Density ρ [kg/m^3^]	2700	200	0
Young’s Modulus E [MPa]	70,000	249.35	0.00248
Poisson’s Ratio [‒]	0.33	279.98	0.0598

**Table 2:** Basic parameters of ALPORAS aluminum foam [37].

	Density [kg/m^3^]	Young’s Modulus [MPa]	Poisson’s Ratio [‒]	Plastic Poisson Ratio *ν_p_* [‒]	Compression Yield Stress Ratio *p*^0^*_c_* [MPa]
**AL**	292	60	0.33	0.015	1.702

**Table 3:** Crashworthiness indicator of aluminum foam-filled columns with different trigger.

		EA [J]	U Max [mm]	PCF [kN]	MCF [kN]	CLE [‒]	SE [‒]	TE [‒]
C	E	1707.6	122.96	37.50	13.89	0.370	0.615	0.228
	AL.-40	1708.2	124.00	36.78	13.78	0.375	0.620	0.232
	AL.-60	1701.8	105.61	37.24	16.11	0.433	0.528	0.228
	AL.-80	1702.6	101.52	37.24	16.77	0.450	0.508	0.229
	AL.-100	1703.7	99.65	37.24	17.10	0.459	0.498	0.229
	AL.-120	1703.2	110.47	37.24	15.42	0.414	0.552	0.229
	AL.-140	1703.8	109.77	37.25	15.52	0.417	0.549	0.229
	AL.-160	1702.3	108.33	37.24	15.71	0.422	0.542	0.229
	AL.-180	1706.1	110.30	37.05	15.47	0.418	0.552	0.230
	AL.-200	1703.6	106.41	37.24	16.01	0.430	0.532	0.229
B	E	1703.2	111.06	37.22	15.34	0.412	0.555	0.229
	AL.-40	1698.8	98.80	37.87	17.19	0.454	0.494	0.224
	AL.-60	1707.7	85.42	37.89	19.99	0.528	0.427	0.225
	AL.-80	1712.9	81.24	37.94	21.08	0.556	0.406	0.226
	AL.-100	1714.9	81.34	37.96	21.08	0.555	0.407	0.226
	AL.-120	1715.2	80.84	37.90	21.22	0.560	0.404	0.226
	AL.-140	1714.6	81.00	37.93	21.17	0.558	0.405	0.226
	AL.-160	1715.2	79.24	37.97	21.64	0.570	0.396	0.226
	AL.-180	1714.1	76.93	37.91	22.28	0.588	0.385	0.226
	AL.-200	1715.8	74.46	38.33	23.04	0.601	0.372	0.224
BC	E	1698.0	108.75	25.98	15.61	0.601	0.544	0.327
	AL.-40	1701.3	111.28	26.14	15.29	0.585	0.556	0.325
	AL.-60	1700.3	107.62	26.30	15.80	0.601	0.538	0.323
	AL.-80	1695.7	102.68	26.26	16.51	0.629	0.513	0.323
	AL.-100	1693.6	101.68	26.28	16.66	0.634	0.508	0.322
	AL.-120	1693.6	101.20	26.30	16.74	0.636	0.506	0.322
	AL.-140	1695.0	97.91	26.32	17.31	0.658	0.490	0.322
	AL.-160	1684.4	95.14	26.28	17.71	0.674	0.476	0.320
	AL.-180	1689.9	91.75	26.30	18.42	0.700	0.459	0.321
	AL.-200	1685.4	88.31	26.26	19.09	0.727	0.442	0.321

**Table 4:** Difference between numerical and experimental energy absorption indicator.

	EA [J]	U Max [mm]	PCF [kN]	MCF [kN]	CLE [‒]	SE [‒]	TE [‒]
CC32-3-S2	1692.36	125.50	40.234	13.485	0.3581	0.628	0.225
C32-3-FEM	1707.57	122.96	37.498	13.887	0.3703	0.615	0.228
Difference [%]	0.90	2.02	6.80	2.98	3.41	2.02	1.31
AL._40_S1	1692.36	125.50	38.734	13.485	0.3481	0.628	0.218
AL._40_FEM	1708.20	124.01	36.780	13.780	0.3750	0.620	0.232
Difference [%]	0.94	1.19	5.05	2.19	7.72	1.20	6.20
AL._60_S1	1701.92	121.93	38.182	13.958	0.3656	0.610	0.223
AL._60_FEM	1689.43	115.53	37.105	14.624	0.3941	0.578	0.228
Difference [%]	0.73	5.25	2.82	4.77	7.81	5.25	2.15
AL._80_S1	1702.47	103.24	38.954	16.490	0.4233	0.516	0.219
AL._80_FEM	1702.64	101.52	37.243	16.772	0.4503	0.508	0.229
Difference [%]	0.01	1.67	4.39	1.71	6.38	1.67	4.61
AL._120_S1	1741.28	102.67	36.654	16.027	0.4373	0.513	0.224
AL._120_FEM	1703.23	110.47	37.239	15.417	0.4140	0.552	0.229
Difference [%]	2.19	7.60	1.60	3.80	5.32	7.60	1.88

## Data Availability

Data is contained within the article.
